# Global health education in U.S. Medical schools

**DOI:** 10.1186/1472-6920-13-3

**Published:** 2013-01-18

**Authors:** Omar A Khan, Richard Guerrant, James Sanders, Charles Carpenter, Margaret Spottswood, David S Jones, Cliff O’Callahan, Timothy F Brewer, Jeffrey F Markuns, Stephen Gillam, Joseph O’Neill, Neal Nathanson, Stephen Wright

**Affiliations:** 1 Community Health & Preventive Medicine, & Global Health Track, Department of Family & Community Medicine, Christiana Care Health System; Global Health Working Group, Delaware Health Sciences Alliance, Wilmington, Delaware, USA; 2 Center for Global Health, International Medicine in the Division of Infectious Diseases and International Health, University of Virginia, Virginia, USA; 3 Department of Family & Community Medicine, Medical College of Wisconsin, International Community Medicine Track, Columbia St Mary’s Family Medicine Residency Program, Milwaukee, WI, USA; 4 Lifespan/Tufts/Brown Center for AIDS Research, Brown University, Providence, USA; 5 Adult Psychiatry Residency Training Program, Cambridge Health Alliance/Harvard Medical School, 1493 Cambridge Street, Cambridge, MA, 02139, USA; 6 Culture of Medicine, Faculty of Arts and Sciences and the Faculty of Medicine, Harvard University, Science Center 371, Oxford St, Cambridge, MA, 02138, USA; 7 Department of Pediatrics, University of Connecticut, Middlesex Hospital Family Medicine Residency, Connecticut, Middletown, USA; 8 Global Health Programs; Department of Medicine, McGill University Medical School, 1020 Pine Ave West, Room 42, Montreal, QC, H3A 1A2, Canada; 9 Department of Family Medicine, BUFM Global Health Collaborative, Boston University Medical Center, Boston, MA, 02118, USA; 10 Institute of Public Health, Public Health Education, University of Cambridge, Cambridge, CB2 2SR, United Kingdom; 11 Global Health Programs & Dept. of Medicine, University of Maryland School of Medicine, 620 W. Lexington St., 4th floor, Baltimore, MD, 21201, USA; 12 Global Health Programs, University of Pennsylvania School of Medicine, 1007 Blockley Hall, Philadelphia, PA, 19104-6021, USA; 13 London School of Hygiene & Tropical Medicine, London, UK

## Abstract

Interest in global health (GH) among medical students worldwide is measurably increasing. There is a concomitant emphasis on emphasizing globally-relevant health professions education. Through a structured literature review, expert consensus recommendations, and contact with relevant professional organizations, we review the existing state of GH education in US medical schools for which data were available. Several recommendations from professional societies have been developed, along with a renewed emphasis on competencies in global health. The implementation of these recommendations was not observed as being uniform across medical schools, with variation noted in the presence of global health curricula. Recommendations for including GH in medical education are suggested, as well as ways to formalize GH curricula, while providing flexibility for innovation and adaptation

## Introduction

Interest in global health (GH) among US medical students is increasing rapidly; quantitative and qualitative data bear out this observation [1-4]. Recent work by expert panels emphasizes that education in globally-relevant health issues should form a basis of contemporary health professions education [5]. It is thus important to review the existing state of GH education for medical students, and suggest future avenues of development.

This paper reviews global health education in US medical schools by levels of comprehensiveness and academic rigor. All available GH recommendations since 1990 are reviewed and their abstracted recommendations presented. This represents the first such comprehensive review, including experts from the US, UK, and Canada, to analyse these data and provide recommendations on the relationship between global health and medical education. These recommendations include ways to formalize GH curricula, while providing enough flexibility for innovation and adaptation.

## Methods

The presence of (1) didactic courses in global health and (2) substantive opportunities to study abroad were used to assess the current state of GH education at US medical schools. A standard literature review was conducted via PubMed databases, web-browsing for search terms, and personal inquiries by the authors into sources of unpublished information. Terms used were combinations of “global”, “international”, “health”, “medical”, “medicine”, “education”, “curriculum”, and “curricula”.

Reports from working groups, councils, and membership organizations were reviewed. The Global Health Education Consortium (GHEC, now part of the Consortium of Universities for Global Health- CUGH) was contacted directly. The Association of American Medical Colleges’ International Opportunities in Medical Education (IOME) database and the Foundation for the Advancement of International Medical Education and Research’s database were accessed as well.

Curricular offerings of 96 US medical schools were selected by participation in the IOME survey [6]. They were evaluated for GH interest group presence, didactic courses in GH, and a close institutional affiliation and/or structured abroad opportunities through the medical school.

Medical schools’ GH development was stratified as follows, with the recognition that the categories are not mutually exclusive:

1) Having a recognized student organization focusing on global or international health.

2) Providing didactic courses in global health, in the basic science or clinical years, either as part of the core curriculum or as a didactic, classroom elective.

3) Close institutional affiliation and/or structured international elective.

Simply allowing students to travel or take courses abroad at institutions unaffiliated with the medical school did not qualify a school for inclusion in either level. For the GH curriculum to be evaluated, there needed to be some commitment by the school to engage with the abroad site; examples of this are discussed in the paper.

For clarification concerning student global health interest groups and global health curricula, some student leaders and course directors were contacted directly.

## Results

The results of the literature review component as well as the research component follow.

### A. Review of existing state of GH education at medical schools

Interest in global health among medical students has increased dramatically, as evidenced by participation in international electives increasing from 6.4% in 1984 [2] to 23.1% in 2007 [1]. Matriculating medical students increasingly have prior international experiences and 20–30% of medical students go overseas [7-9].

**Figure 1:**
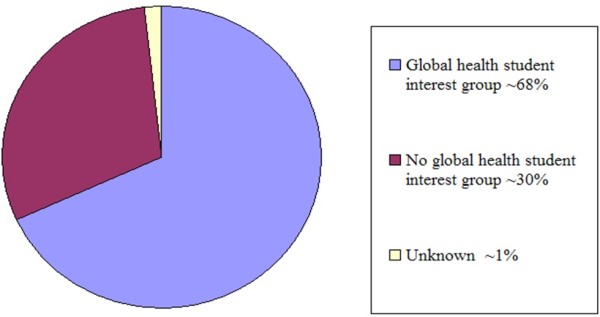
Global health student interest group presence in medical schools.

Of 116 United States allopathic schools surveyed in 2010, 79 (68%) had active student global or international health interest groups while 35 (30%) did not; data were not obtained from 2 schools “See Figure 1”.

Schools were evaluated to determine if they reported any GH offerings on the IOME survey [6]. Opportunities included pre-travel preparatory courses, language/cultural immersion, pre-clinical summer opportunities, clinical/research electives, and/or a global health track. The medical schools could have provided these opportunities themselves or via partnerships with others. About 90% of schools self-reported as offering a global health opportunity for students. Schools were also evaluated for offering didactic coursework in global health; 39% of schools did so. Finally, schools were evaluated for offering a substantive abroad experience for students; 47% of schools surveyed offered such an experience.

### B. Review of existing recommendations for GH curricula [see Table 1]

**Table 1:** Review of existing recommendations for GH curricula


**A.** In 1990, “A National Consensus on the Essential International-health Curriculum for Medical Schools” was developed [2]. That study, focusing on medical student education, polled twenty-two International Health Medical Education Consortium (now the Global Health Education Consortium- GHEC) members and concluded:	
1.	Curricula vary based upon the location abroad
2.	Clinical experiences form the bulk of rotations abroad; preparation via case studies and problem-based learning is optimal
3.	Community-based primary health care should be a core component of the preparatory curriculum
4.	Abroad opportunities can expand the physician role to assessment and management of community health programs and to train the team’s paramedical members
5.	Interdisciplinary faculty teams can often successfully teach global health courses.
**B.** Since then, GHEC has merged with the Consortium of Universities for Global Health (CUGH); the latter was created to coordinate GH efforts across institutions [12] and identified many deficiencies with GH education [13], and put forward the following recommendations:	
1.	Medicine and public health must respond to changing conditions as a result of advances and innovations in technology, an increased focus on human and civil rights, globalization, and the growing passion among students, faculty and professionals to address global health.
2.	The emerging discipline of global health must be defined, reflecting major global health challenges with a focus on “interdependence;” including disciplines beyond health to include law, engineering, agriculture, social sciences and business.
3.	Make the academic enterprise a transforming agent in global health, recapturing the University as part of the community, not an “ivory tower.” Ensure that academic training in global health emphasizes capacity building and the training of leaders and managers.
4.	Expand academic exchange programs through mutually beneficial “academic twinning” between academic institutions in the developed and developing countries.
5.	Address the “brain drain” problem and the strategic ways it might be managed.
6.	Develop research capacity in developing countries, emphasizing the “Bench-to-Burkina Faso” principle, i.e., translate discovery to implementation.
**C.** The Association of Faculties of Medicine of Canada Resource Group on Global Health/GHEC joint committee partnership proposed, in 2005, seven possible areas of essential global health knowledge: 1) human rights, 2) the social determinants of health, 3) policy, trade, and health, 4) the global burden of disease, 5) health care delivery systems, 6) the environment and health and 7) migration, travel, and global interaction [14]. That has been updated to the most recent (2010) guidelines which propose that a medical graduate should have competency in the following areas:	
1.	Global Burden of Disease
2.	Health implications of travel, migration and displacement
3.	Social and economic determinants of health
4.	Population, resources and environment
5.	Globalization of health and healthcare
6.	Healthcare in low-resource settings
7	Human rights in global health [14,63]
**D.** In 2007, Houpt et al recommended the Liaison Committee on Medical Education (LCME) establish a thirty hour standard curriculum in global health as a necessary minimum for future physicians to be competent to treat changing populations [1]. This group’s definition of Global Health was “the global commonality of health issues that transcend national borders, class, race, ethnicity, income, or culture.” Some examples of such global health issues include poverty, limited access to health care, status of women, environmental degradation, political instability, war, genetic susceptibility, and the experience of industrialization which can lead to chronic health issues. Thus, Houpt et al concluded that the distinction between domestic and international health problems is no longer useful.	
**E.** In 2006, competency domains for medical students’ GH experiences were proposed by Eckhert et al as follows:	
1)	Medical knowledge of international diseases
2)	Review of basic history and physical exam skills augmented by a need to apply old skills in a dissimilar setting
3)	Cultural sensitivity
4)	Educational preparation (objectives, responsibilities, supervision)
5)	Quantifying success “encouraging students to think more broadly and see the patient in the context of his or her community or even the world” [8].
**F.** GH learning outcomes for UK medical students were developed by the UK Global Health Learning Outcomes Working Group [15]. A similar competency-based approach was reviewed by Battat et al [64].	
1)	Global burden of disease
2)	Socioeconomic and environmental determinants of health
3)	Health systems
4)	Global health governance
5)	Human rights and ethics
6)	Cultural diversity and health

We searched for both ‘international health’ and ‘global health’ curricula. A fuller discussion of the distinction between the terms is outside the scope of this paper, as it has been discussed in recent work elsewhere [10]. There has been a shift away from the former to the latter term, which will be used in this paper [11].

Recommendations from the following are discussed in more detail in the Table 1:

A. “A National Consensus on the Essential International-health Curriculum for Medical Schools”, from 1993 [2].

B. The Consortium of Universities for Global Health (CUGH) [12,13].

C. The Association of Faculties of Medicine of Canada Resource Group on Global Health/GHEC joint committee [14].

D. The recommendations from Houpt et al [1].

E. Domains of competency in GH by Eckert at al [8].

F. GH learning outcomes for UK medical students, developed by the UK Global Health Learning Outcomes Working Group [15,16].

## Discussion

This is the first time global health curricula in US medical schools have been systematically described, and the current recommendations for GH education reviewed. GH student interest groups were used as a proxy for medical student interest in the field. The findings indicate that this interest is uniformly high across all types of medical schools. The demand for global health education is demonstrably rising, as is university-level engagement [17].

We agree in the main with Houpt et al. that the distinction between domestic and international health problems may no longer be a useful one [1]. Since then, additional work supports this viewpoint across the health sciences [5,10].

We did not find uniformity in the way medical school curricula in the US followed published GH guidelines. A recent review undertook a similar survey of GH in Canada [18]; while it did not assess the quality of education nor provide recommendations for GH education, it also found little standardization of GH curricula in Canadian medical schools.

While many studies were observational in nature and did not provide control groups, several demonstrated benefits of medical trainees’ exposure to GH education. Older data indicate higher scores on the USMLE board exam for those with GH exposure [19]; however, the demonstrated value of a GH curriculum probably cannot, and perhaps should not, be measured solely by test results. A more likely benefit is increased awareness of the role of public health in medicine and greater awareness of social and economic barriers to patient care [20,21].

No published studies explored these issues among medical students in a rigorous (i.e. randomized prospective or case–control) fashion. However, the studies reviewed, as well as our collective experience of decades of GH education, suggest that trainees return with a greater awareness of the many factors that affect the health of individuals and populations, in the US or abroad [21,22].

This review suggests that among those with GH exposure as students, there is an increased chance of future practice in underserved areas of the United States, as well as in primary care, or both [20,23]. This mirrors the observation from Europe that those with GH training are more likely to work in rural practices, in primary care [24]. It remains unclear whether this is a result of a self-selection, i.e. those already inclined towards this type of practice chose to go abroad during medical school, rather than a greater appreciation of underserved health due to their GH experiences. It is also unclear whether the desire to work in underserved areas is sustained over time.

This correlation has also been found in studies of Canadian GH programs in medical schools, and they are equally cautious in ascribing primary care motivations to the GH experiences alone [18]. However, such an association may be useful in maximizing this inclination through GH and other experiences.

We found that more schools (47%) provided an abroad experience than a didactic one locally (39%). This may be due to the challenge of freeing up clinical faculty time to teach classroom courses. In our view, the provision of both is preferable to each one by itself, since it allows the linkage of theoretical and practical information, conforming to the template for undergraduate medical education in general.

### Recommendations

#### Development of curricular guidance

We favor core competencies applicable to global health for (a) medical students; (b) physicians working in a significant health-related capacity outside the US. Many if not most of these competencies should be applicable to underserved communities in the US as well, and are mirrored in UK guidelines as well [16].

Growing student interest in GH is an opportunity for US medical school educators. Student interest in GH can be harnessed to teach them principles of preventive medicine and public health which would help in virtually any patient- or population-based field, and applies to primary care and specialty training.

It has been recommended that the LCME establish a standard curriculum in global health as a necessary minimum [1]. However, there is no convincing evidence that a standard 30-hour curriculum provides competency in the scope or complexity of global health. Indeed, such training may take the form of a full residency in countries such as the UK, before a physician can be considered competent in tropical medicine [25]. As mentioned earlier, unlike US schools of public health, which have well-defined global health curricula [26], there is presently a lack of uniformity in such offerings in US and Canadian medical schools [27]. As the field of GH in medicine matures, there may initially need to be a balance between prescriptiveness and libertarianism. There is general agreement on the concepts of GH competencies, sharing of lessons learned, and collaborations, in medical school and residency [28-32]. However, this consensus stops short of a standardized, testable GH curriculum for all medical schools, although some experts have issued a call for standardization on the definition of global health and GH education [10,28]. Similar to how the Flexner report brought coherence to medical education, so too should the foundations of GH build from an evidence-based core [5,33-35].

As individual educators, we have been challenged with providing a baseline level of GH knowledge to all students or to tailor a curriculum to the self-selected few. The solution may be a basic curriculum applicable to all medical students, with progressively more advanced electives available, including a Master’s in Public Health (MPH) or similar degree, where applicable. Based on their resources and interest, schools may choose to adopt a high-intensity model incorporating many levels targeting students of varying motivation, or a lower-intensity model providing a baseline level of GH topics to all medical students, or a combination of both.

Contemporary GH thinking requires sustainable, systems-based approaches to solutions, rather than reductive interventions. GH competencies should, at the minimum, include the recognition of an epidemiologic transition, such that the dual burden of infectious and chronic diseases now threaten the developing world [36].

While traditional ‘tropical diseases’ such as malaria (and newer diseases affecting the developing world such as HIV/AIDS) should continue to be taught, the emphasis should be on educational elements applicable universally. Over the last decade, the concept of social determinants of health (SDH) has been recognized as critical to GH worldwide and disproportionately so in the developing world [37,38]. A foundational education in GH including principles of epidemiology, burden of disease and SDH is thus far more likely to equip the US medical student with critical thinking skills applicable abroad or in the US, compared with a curriculum focusing solely on diseases/treatments of the tropics.

As one example of a layered model, the global health curriculum at the University of Vermont College of Medicine provides a baseline level of GH education to all medical students, via introductory lectures at Orientation; matching with a global health-oriented faculty member on request; and a ‘Bridge’ curriculum in GH between the clerkship and senior year of medical school. A didactic 1-month elective in global health is available to all seniors, as is a 1-month abroad elective at one of two partner sites in Bangladesh, with an equal emphasis on development of the host center [39]. Each year, approximately 15–20 students (18-25% of the class) opt for the didactic elective, and 3–5 for the experiential, abroad elective. Each year for the last 3 years, at least one student has pursued an MPH degree for further training in global and community health.

Within didactic electives, there remains a need for a textbook of GH in medicine. There are many introductory textbooks of global public health available, some with readings suitable for an introductory course on GH in medical school [40-44]. Some are essentially field manuals for tropical medicine diagnosis and treatment [45,46]. None, however, is aimed primarily at a comprehensive GH approach for the clinical sciences student. A defining textbook would allow all medical schools (despite differing levels of expertise among the faculty) access to a standard source of rigorous curricular information in global health. The optimum solution may be to supplement a standardized set of readings with a robust online component of updated information, updates, and supplementary material.

### Engaging ethically with global partners

In the case of global health electives abroad, there should be clear expectations for a “best practice” relationship with a host institution. While such models have been described [39], many in the developing world remain vulnerable to exploitation by more powerful interests, within and beyond their borders [47-49].

There is not only a burden placed upon host institutions and communities by having visiting learners, but also the ethical and moral imperative to conduct clinical experiences with the same expectations as US-based work, regarding supervision and extent of involvement in patient care. Such concerns may seem archaic but evidence of exploitation continues to surface [50-52]; relatively recent accounts of inappropriate use of trainees abroad [49] make it imperative that the conduct of US trainees and their faculty be above reproach.

It is important to define the student role to be observational or participatory, and if the latter, to what extent. Any work undertaken should be at the level of training assessed by their home (US) medical school. In general, procedures they would not be allowed to do in the US would be similarly restricted abroad. Ethical guidelines for global health work have been articulated and are available [53-56]. Furthermore, the role of students in such work has specifically been addressed [57,58]; these guidelines should be adapted for inclusion in all GH curricula.

There are other issues related to international partnerships which are beyond the scope of this paper; these include the debate over ‘brain drain’, e.g., whether the effect that bringing trainees to the US would have on the developing/donor country [59,60]. Twinning programs between two (or more) partners have the potential to achieve educational goals in an ethical fashion. Recent work by the Commission on Education of Health Professionals for the 21^st^ Century has emphasized this and pointed to the success of such programs in Kenya, Nigeria and Uganda, twinned with counterpart institutions in the UK and North America [5].

While ethical engagement is understood by most as an important need, the related issue of reciprocity often comes up. This is particularly relevant to medicine for two reasons. First, while heeding the dictum of *primum non nocere* (L. *first do no harm*) is essential, it is but the bare minimum. Simply doing no harm does not excuse us from the need to actually do good. Second, unlike counterparts in public health who regularly engage with developing countries while conducting research and training programs, clinical medicine has many fewer examples of sustainable medical partnership programs. As medical professionals engage further with global health and with counterparts in public health, there is an opportunity for shared learning.

### The need for leadership involvement

In order to promote and advocate for GH within and without, these initiatives need support from the highest levels of the university. GH in US medical schools is belatedly benefiting from leadership involvement. At the second meeting of the CUGH in September 2010, a roundtable included eight presidents of member universities, indicating their present and ongoing commitment to GH education [12]; such a commitment is a pre-requisite to membership in the organization.

There is a risk management aspect to this: when sending medical students abroad, university/hospital legal departments may need to be involved due to issues of risk, liability, health, and airlifting policies. These risks can be managed, but if they are overstated, the GH initiatives themselves may come under question. In order to promote and advocate for GH within and without, such initiatives need support from the highest levels of the university.

It is our recommendation that medical institutions involved in GH education should have university-wide participation, and the endorsement of the university president and medical school dean. Abroad initiatives should also involve leadership of the counterpart institution. Reasons for leadership support include the need for political will to support an initiative without (initially) an obvious clinical funding stream. They may also support the need for protected faculty time to teach, work on grants for GH, engage in elective management, and build/maintain sustainable relationships with partners.

Some US health centers have launched university-wide initiatives such as centers, institutes or even departments (and in some cases, across more than one institution) devoted to GH [61,62]. The exact institutional structure, however, is less important than establishing productive funding streams and collaborations -- within and between universities, and with global educational partners -- for successful global health education, research, and practice.

## Conclusion

The significant, rapid growth in interest regarding global health at US medical schools seems at present to be outpacing the development of standardized curricula. The current environment of somewhat fragmented curricular development is gradually transitioning to increased collaboration, emergence of best practices and shared models. GH tracks in residency and medical school are taking advantage of this. Our group is among the first to evaluate this topic among medical students in a systematic fashion. Global health represents exciting opportunities for teachers and learners alike; it is essential we think carefully through these and respond thoughtfully with evidence-based guidance.

## Pre-publication history

The pre-publication history for this paper can be accessed here:

http://www.biomedcentral.com/1472-6920/13/3/prepub
